# Comparison of the long-term efficacy between entecavir and tenofovir in treatment- naïve chronic hepatitis B patients

**DOI:** 10.1186/s12876-017-0596-7

**Published:** 2017-03-09

**Authors:** Ji Won Park, Kyeong Min Kwak, Sung Eun Kim, Myoung Kuk Jang, Ki Tae Suk, Dong Joon Kim, Sang Hoon Park, Myung Seok Lee, Hyoung Su Kim, Choong Kee Park

**Affiliations:** 1 Department of Internal Medicine, Hallym University Sacred Heart Hospital of Hallym University Medical Center, 22, Gwanpyeong-ro 170 beon-gil, Dongan-gu, Anyang-si, Gyeonggi-do 14068 Republic of Korea; 2 Department of Biomedical Gerontology, Graduate School of Hallym University, 1 Hallymdaehak-gil, Chuncheon-si, Gangwon-do 24252 Republic of Korea; 3 Department of Occupational and Environmental Medicine, Hallym University Sacred Heart Hospital of Hallym University Medical Center, 22, Gwanpyeong-ro 170 beon-gil, Dongan-gu, Anyang-si, Gyeonggi-do 14068 Republic of Korea; 4 Department of Environmental Health, Seoul National University School of Public Health, 1, Gwanak-ro, Gwanak-gu, Seoul, 08826 Republic of Korea; 5 Department of Internal Medicine, Kangdong Sacred Heart Hospital of Hallym University Medical Center, 18, Cheonho-daero 173-gil, Gangdong-gu, Seoul 05355 Republic of Korea; 6 Department of Internal Medicine, Chuncheon Sacred Heart Hospital of Hallym University Medical Center, 77, Sakju-ro, Chuncheon-si, Gangwon-do 24253 Republic of Korea; 7 Department of Internal Medicine, Kangnam Sacred Heart Hospital of Hallym University Medical Center, 1, Singil-ro, Yeongdeungpo-gu, Seoul, 07441 Republic of Korea

**Keywords:** Entecavir, Tenofovir, Hepatitis B virus

## Abstract

**Background:**

There have been limited studies directly comparing the long-term efficacy between entecavir (ETV) and tenofovir disoproxil fumarate (TDF). This study was aimed to compare the long-term efficacy between them in treatment-naïve chronic hepatitis B (CHB).

**Methods:**

Out of 345 CHB patients who received first line therapy with ETV (*n* = 200) or TDF (*n* = 145) in a cohort, 210 patients were analyzed using propensity score matching, at a ratio of 1:1.

**Results:**

Two groups showed no difference in baseline characteristics. During the follow-up of 12 months, HBV DNA levels were similarly suppressed in both groups (ETV vs. TDF; −5.01 vs. -5.242 log_10_IU/mL, *P* = 0.559). At month 12, both groups showed no difference in terms of the serologic, biochemical and virologic (VR) response. In multivariate analysis, the initial virologic response at 3 months (IVR-3) was independent factor for VR at 1 year. During the long-term follow-up, HBV DNA levels were more strongly suppressed by TDF than ETV in hepatitis B e antigen (HBeAg) positive patients (*P* = 0.035), especially with high viral load (*P* = 0.012), although there was no significant difference in overall VR between two groups. The type of antivirals was not an independent factor for long-term VR.

**Conclusions:**

Although either ETV or TDF, overall, may show a comparable long-term antiviral efficacy in treatment-naïve CHB, TDF might be better regimen than ETV in the subgroup of HBeAg-positive CHB, especially with a higher HBV DNA levels.

## Background

Approximately 350 million people are estimated to be chronically infected with hepatitis B virus (HBV), which may result in serious complications, such as hepatic failure, advanced cirrhosis, and/or hepatocellular carcinoma (HCC) in 15–40% of patients [1–3]. In addition to clinical host factors, such as age, family history, and alcohol abuse, many HBV-related factors themselves, which include HBV mutations of precore/core promoter regions, HBV genotype C, and high serum HBV DNA levels (viral factors), contribute to the critical progression of chronic liver diseases [4–6]. Although factors such as mutations or genotypes are not changed by antivirals, serum HBV DNA levels, e.g., HBV replication, can be sustainably suppressed by anti-HBV therapies to prevent and/or retard cirrhosis and subsequently HCC. To date, oral antiviral drugs such as lamivudine, adefovir, entecavir, telbivudine and tenofovir have been widely used in clinical settings [4, 7, 8].

According to several international guidelines, entecavir (ETV) or tenofovir disoproxil fumarate (TDF) are recommended as first line therapy in the treatment of naïve CHB because of their higher antiviral potency and higher genetic barriers than other antiviral agents [9–13]. In reality, ETV and TDF showed high virologic responses of up to 93% and 100%, respectively, and a rare genotypic resistance of only 1.2% and 0%, respectively, during the 5- year follow-up in a cohort study [14, 15].

Although there have been a few reports comparing the short-term efficacy directly between TDF and ETV in treatment-naïve CHB, there is still little available clinical information about the long-term efficacy. Therefore, we performed this study to compare the long-term efficacy between TDF and ETV in treatment-naïve CHB patients.

## Methods

### Patients and study design

A total of 345 treatment-naïve CHB patients who received a single regimen of either TDF (*n* = 145) or ETV (*n* = 200) for at least 12 months were consecutively enrolled from 4 tertiary university hospitals in Korea between January 2011 and December 2014. Because TDF or ETV use was not randomly assigned, potential confounding and selection biases were accounted for by developing a propensity score. Among a total of 345 patients, 210 patients were selected using propensity score match, at a ratio of 1:1. Patients with the following characteristics were excluded: 1) other viral infections such as HCV, HDV and HIV, 2) other concomitant liver diseases such as alcoholic liver disease and autoimmune liver disease, or 3) HCC. Patients were monitored by clinical examination and biochemical and virologic assessments at least every 3 to 4 months during the antiviral therapy.

The study protocol was approved by the Hallym University Medical Center Institutional Review Boards (IRB No. 13-1-49).

### Serum assay and methodology

Biochemistry was performed using standard laboratory procedures. Hepatitis B surface antigen (HBsAg), antibody to HBsAg (anti-HBs), HBeAg, and antibody to HBeAg (anti-HBe) were measured using microparticle enzyme immunoassay (Abbott Laboratories, North Chicago, IL, USA). Serum HBV DNA levels were measured using a COBAS TaqMan PCR assay (Roche, Branchburg, NJ, USA; lower limit of detection 20 IU/mL).

### Definitions

Primary non-response was defined as a failure to reduce serum HBV DNA levels by >1 log_10_ IU/mL after 3 months of therapy [16]. A high HBV DNA level was defined as a serum HBV DNA level greater than 6 log_10_ IU/mL. Initial virologic response at 3 months (IVR-3) and virologic response (VR) were defined as an HBV DNA level <3.3 log_10_ IU/mL after 3 months of therapy [17, 18] and an undetectable HBV DNA level (<20 IU/mL) during the therapy, respectively. A biochemical response was defined as a normalization of the serum alanine aminotransferase (ALT) levels. Virologic breakthrough (VBT) was defined as an increase over 1 log_10_ IU/mL of serum HBV DNA levels from the nadir during the therapy or serum HBV DNA levels >200 IU/mL in patients who experienced VR.

### Statistical analyses

HBV DNA levels were logarithmically transformed for analysis. The Mann–Whitney *U*-test for continuous variables and chi-squared test for categorical variables were used in the analyses as appropriate. A repeated measures analysis was used to compare HBV DNA reduction according to the drug used. Cumulative VR and VBT rates were calculated using the Kaplan-Meier method, and the difference was determined by a log-rank test. The multivariate logistic regression model and Cox proportional hazards model were used to identify the independent risk factors significantly associated with short-term and long-term VR, respectively. Candidate variables with a *P* value of < 0.1 on univariate analysis were entered into the regression analysis. A *P*-value of less than 0.05 was considered significant. Statistical analyses were performed using SPSS, version 16 (SPSS, Inc, Chicago, IL).

To reduce and control any confounding factors before receiving the treatment, we used the propensity scores to match TDF users to ETV users. A macro (available at: http://www2.sas.com/proceedings/sugi26/p214-26.pdf) was used to develop the propensity scores.

## Results

### Baseline patient characteristics

Among 345 patients, the propensity-matched 105 patients in each group were included (1:1). The baseline characteristics of the study population are summarized in Table 1.

**Table 1:** Baseline characteristics of the patients

Variables	ETV (*n* = 105)	TDF (*n* = 105)	*P*
Age (years)	47.0 ± 12.0	45.5 ± 11.9	0.396
Sex (male, %)	64 (61.0)	67 (63.8)	0.776
HBeAg-positive (%)	58 (55.2)	63 (50.0)	0.577
Disease status; CHB/LC (%)	62/43 (59.0/41.0)	64/41 (61.0/39.0)	0.888
MELD (<7/7-13/>13, %)	73/26/6 (69.5/24.8/5.7)	71/33/1 (67.6/31.4/1.0)	0.800
Serum ALT (IU/L)	179.5 ± 253.0	232.3 ± 427.7	0.278
Serum total bilirubin (mg/dL)	1.20 ± 1.74	1.06 ± 1.29	0.507
Serum albumin (g/dL)	4.11 ± 0.56	4.10 ± 0.53	0.905
INR	1.12 ± 0.19	1.11 ± 0.17	0.927
Serum HBV DNA level (log_10_ IU/mL)	6.56 ± 1.33	6.66 ± 1.26	0.564
Duration of follow-up (months)	27.0 ± 7.2	23.6 ± 5.2	

Continuous variables were expressed as mean ± standard deviation

*ALT* alanine aminotransferase, *CHB* chronic hepatitis B, *ETV* entecavir, *HBeAg* hepatitis B e antigen, *HBV* hepatitis B virus, *INR* international normalized ratio, *LC* liver cirrhosis, *MELD* model for end stage liver disease, *TDF* tenofovir

One hundred thirty one (62.4%) patients were men and the mean age was 46.2 ± 12.0 years. Eighty four patients (40%) had had cirrhosis and 121 patients (57.6%) were positive for HBeAg. The mean HBV DNA levels and ALT levels were 6.61 ± 1.30 log_10_ IU/mL and 205.9 ± 351.5 IU/L, respectively. There was no difference in the baseline characteristics between the TDF and ETV groups. On average, patients were treated for 25.3 (12–39) months.

### Therapeutic responses

**Fig. 1:**
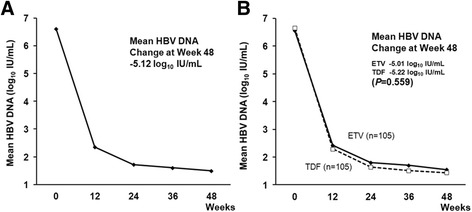
Change of HBV DNA during follow-up period. **a** The changes in the mean hepatitis B virus (HBV) DNA levels from baseline through week 48. The overall mean changes at weeks 12, 24 and 48 were −4.26 log_10_ IU/mL, −4.89 log_10_ IU/mL and −5.12 log_10_ IU/mL, respectively. **b** The mean reduction in serum HBV DNA levels from baseline to week 48 was similar in tenofovir and entecavir group (−5.22 vs. −5.01 log_10_ IU/mL, *P* = 0.559) by repeated measure analysis

Figure 1 shows the changes in the mean HBV DNA levels during the first 12 months. Overall, serum HBV DNA levels continuously declined, and the overall mean changes at months 3, 6 and 12 were −4.26 log_10_ IU/mL, −4.89 log_10_ IU/mL and −5.12 log_10_ IU/mL, respectively (Fig. 1a). The mean reduction in serum HBV DNA levels from baseline to month 12 was similar in the ETV and TDF groups (−5.01 vs.−5.22 log_10_ IU/mL, *P* = 0.559) using a repeated measure analysis (Fig. 1b). During the first year of therapy, VR (HBV DNA levels < 20 IU/mL) and primary non-response were observed in 161 (76.7%) and 0 patients (0%), respectively. One hundred fifty of 184 patients who had shown elevated serum ALT levels at baseline achieved a normalization of serum ALT levels (81.5%). During the first year of therapy, HBeAg loss/seroconversion occurred in 18 of 121 (14.9%) HBeAg-positive patients.

**Fig. 2:**
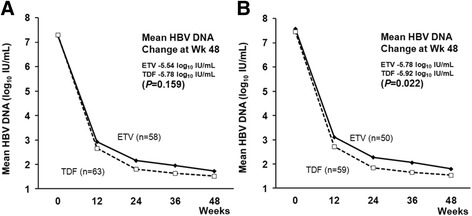
Change of HBV DNA in HBeAg-positive patients. **a** The mean reduction in serum hepatitis B virus (HBV) DNA levels from baseline to month 12 was similar in tenofovir and entecavir group (−5.88 vs. −5.54 log_10_ IU/mL, *P* = 0.159) in the hepatitis e antigen (HBeAg) positive patients. **b** Serum HBV DNA levels were more strongly suppressed by tenofovir than entecavir (−5.92 vs. −5.78 log_10_ IU/mL, *P* = 0.022) in the HBeAg-positive patients with a higher HBV DNA levels

There were no significant differences in the virologic, serologic and biochemical responses at year 1 between two groups (Table 2). The mean reduction in serum HBV DNA levels from baseline to month 12 was similar between the ETV and TDF groups (−5.54 vs.−5.78 log_10_ IU/mL, *P* = 0.159), even in the HBeAg-positive patients (Fig. 2a). Although HBV DNA levels were more strongly suppressed by TDF than by ETV (−5.92 vs. −5.78 log_10_ IU/mL, *P* = 0.022) in the HBeAg-positive subgroup with higher HBV DNA levels (Fig. 2b), the type of antiviral used was not an independent factor for VR. During the mean long-term therapy period of 25.3 months, the virologic and serologic responses were additionally achieved in 22 and 10 patients, respectively. Persistent viremia was observed in 26 patients, consisting of 17 patients (16.2%, 17/105) in the ETV group and 9 patients (8.6%, 9/105) in the TDF group.

**Table 2:** Virologic, serologic and biochemical response at week 48

	Baseline	Week 24	Week 48	Total	*P*
Mean HBV DNA (log_10_ IU/mL)	6.61	1.72	1.49	5.12↓	0.559
ETV (*n* = 105)	6.56	1.80	1.55	5.01↓	
TDF (*n* = 105)	6.66	1.64	1.44	5.22↓	
HBV DNA negativity by PCR				161(76.7%)	0.192
ETV (*n* = 105)		58 (55.2%)	76 (72.4%)		
TDF (*n* = 105)		56 (53.3%)	85 (81.0%)		
HBeAg loss/seroconversion				18 (14.9%)	0.452
ETV (*n* = 58)			7 (12.1%)		
TDF (*n* = 63)			11 (17.5%)		
Biochemical response				150 (81.5%)	0.647
ETV (*n* = 92)			77 (83.7%)		
TDF (*n* = 92)			73 (79.3%)		

Data are expressed mean or number

*ETV* entecavir, *HBeAg* hepatitis B e antigen, *HBV* hepatitis B virus, *TDF* tenofovir

**Fig. 3:**
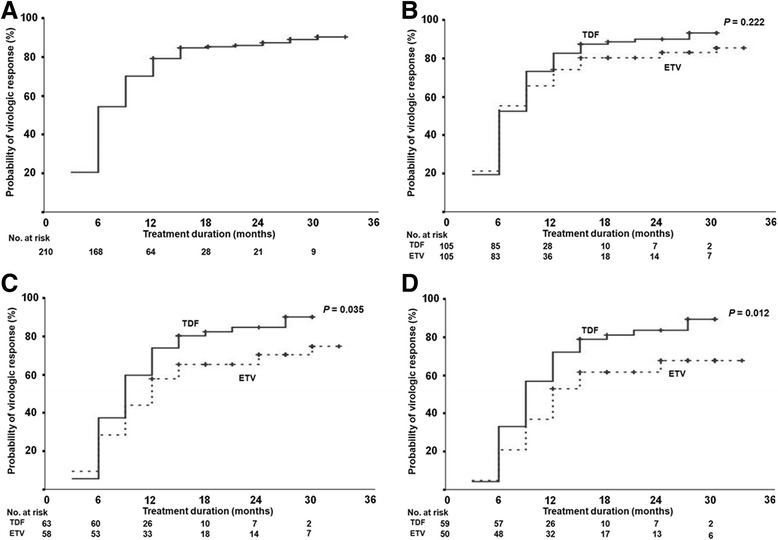
Cumulative virologic response (VR) rate. **a** Cumulative VR rates at 3, 6, 12, 24 and 36 months were 20%, 54%, 79%, 86% and 91%, respectively. **b** Cumulative VR rates were not significant different between the two groups (*P* = 0.222). **c** Cumulative VR rates of tenofovir group were higher than those of entecavir group, in the hepatitis B e antigen (HBeAg) positive patients. **d** The difference of cumulative VR rates was more evident in HBeAg-positive patients with a higher hepatitis B virus DNA levels (>6 log_10_ IU/mL)

The cumulative VR rates at 3, 6, 12, 24 and 36 months were 20%, 54%, 79%, 86% and 91%, respectively, during the follow-up period (Fig. 3a). The cumulative VR rates in the ETV group at 3, 6, 12, 24 and 36 months were 21%, 55%, 75%, 84% and 87%, respectively, whereas, in the TDF group, the rates at 3, 6, 12, 24 and 36 months were 19%, 52%, 83%, 91% and 94%, respectively. These differences were not significant (Fig. 3b, *P* = 0.222). The cumulative VR rates in the HBeAg-negative subgroup also showed no significant difference between the ETV and TDF groups (89%, 96% and 100% vs. 76%, 98% and 100% at 6, 12 and 24 months, respectively, *P* = 0.819). However, in the HBeAg-positive subgroup, the cumulative VR rates in the ETV group at 6, 12, 24 and 36 months were 28%, 57%, 70% and 77%, respectively, whereas the rates in the TDF group at 6, 12, 24 and 36 months were 37%, 73%, 84% and 91%, respectively, with significant difference between two groups (*P* = 0.035, Fig. 3c). This finding was conspicuous in another subgroup of HBeAg-positive CHB with higher HBV DNA levels (20%, 53%, 68% and 68% at 6, 12, 24 and 36 months in ETV group vs. 32%, 71%, 83% and 90% at 6, 12, 24 and 36 months in TDF group, respectively, *P* = 0.012, Fig. 3d).

### Predictive factors for virologic response

A multivariate logistic regression model was used to identify the independent risk factors significantly associated with VR during the first year. In a univariate analysis, disease status (CHB vs. cirrhosis), HBeAg status, serum HBV DNA levels and IVR-3 were candidate variables for the multivariate analysis. Of the clinical features, cirrhosis, HBeAg negativity, low serum HBV DNA levels and IVR-3 were considered favorable factors for VR, while other factors, including the type of antiviral used (ETV vs. TDF), were not significantly associated with VR (Table 3). In the multivariate analysis, only IVR-3 remained independent predictors for one year VR (Table 4).

**Table 3:** Comparison of clinical features between the groups according to one year virologic response

Variables	Patients with VR (*n* = 161)	Patients without VR (*n* = 49)	*P*
Age (years)	46.8 ± 11.0	44.4 ± 14.7	0.285
Sex (male, %)	99 (61.5)	32 (65.3)	0.737
HBeAg-positive (%)	77 (47.8)	44 (89.8)	<0.001
Disease status; CHB/LC (%)	85/76 (52.8/47.2)	41/8 (83.7/16.3)	<0.001
MELD (<7/7-13/>13, %)	109/47/5 (67.7/29.2/3.1)	35/12/2 (71.4/24.5/4.1)	0.768
Serum ALT (IU/L)	198.0 ± 305.7	231.8 ± 475.0	0.557
Serum total bilirubin (mg/dL)	1.12 ± 1.46	1.19 ± 1.77	0.774
Serum albumin(g/dL)	4.10 ± 0.53	4.11 ± 0.60	0.952
INR	1.12 ± 0.17	1.09 ± 0.21	0.319
IVR-3 (−/+)	12/149(7.5/92.5)	25/24 (51.0/49.0)	<0.001
Serum HBV DNA level (log_10_ IU/mL)	6.30 ± 1.23	7.61 ± 0.97	<0.001
Treatment regimens			0.192
ETV (*n* = 105, %)	76 (72.4)	29 (27.6)	
TDF (*n* = 105, %)	85 (81.0)	20 (19.0)	

Data are expressed as mean ± standard deviation or number

*ALT* alanine aminotransferase, *CHB* chronic hepatitis B, *ETV* entecavir, *HBeAg* hepatitis B e antigen, *HBV* hepatitis B virus, *INR* international normalized ratio, *IVR-3* initial virologic response at 3 months, *LC* liver cirrhosis, *MELD* model for end stage liver disease, *TDF* tenofovir, *VR* virologic response

**Table 4:** Multivariate analyses of clinical factors affecting one year virologic response

Factors	RR	95% CI	*P*
Disease status (CHB/LC)	0.559	0.205–1.521	0.255
IVR-3 (+/−)	6.214	2.500–15.443	<0.001
HBeAg (−/+)	3.184	0.977–10.377	0.055
Serum HBV DNA level (log_10_ IU/mL)	0.605	0.357–1.023	0.061

*RR*, relative risk, *95% CI* 95% confidence interval, *CHB* chronic hepatitis B, *HBeAg* hepatitis B e antigen, *HBV* hepatitis B virus, *IVR-3* initial virologic response at 3 months, *LC* liver cirrhosis

A cox proportional hazards model was used to identify the independent factors for long-term VR. In the multivariate analysis, HBeAg status, serum HBV DNA levels and IVR-3 were identified as predictive factors for long-term VR (Table 5).

**Table 5:** Factors associated with long-term virologic response

Factors	Univariate analysis			Multivariate analysis		
	RR	95% CI	*P*	RR	95% CI	*P*
Age (years)	1.007	0.995–1.018	0.262			
Sex (female/male)	1.009	0.747–1.363	0.952			
HBeAg-positive (−/+)	3.031	2.209–4.158	<0.001	1.834	1.246–2.698	0.002
Disease status (LC/CHB)	1.577	1.172–2.121	0.003	1.028	0.748–1.411	0.867
MELD (<7/≥7)	0.923	0.676–1.260	0.613			
INR	1.020	0.469–2.218	0.961			
Serum ALT (IU/L)	1.000	1.000–1.000	0.890			
Serum total bilirubin (mg/dL)	1.005	0.918–1.101	0.908			
Serum albumin (g/dL)	1.077	0.824–1.406	0.588			
IVR-3 (+/−)	4.851	2.902–8.109	<0.001	3.403	1.977–5.859	<0.001
Serum HBV DNA level (log_10_ IU/mL)	0.638	0.569–0.715	<0.001	0.817	0.696–0.958	0.013
Therapy regimens (TDF/ETV)	1.162	0.868–1.555	0.313			

*RR* relative risk, *95% CI* 95% confidence interval, *ALT* alanine aminotransferase, *CHB* chronic hepatitis B, *ETV* entecavir, *HBeAg* hepatitis B e antigen, *HBV* hepatitis B virus, *INR* international normalized ratio, *IVR-3* initial virologic response at 3 months, *LC* liver cirrhosis, *MELD* model for end stage liver disease, *TDF* tenofovir

### Predictive factors on the long-term efficacy

**Fig. 4:**
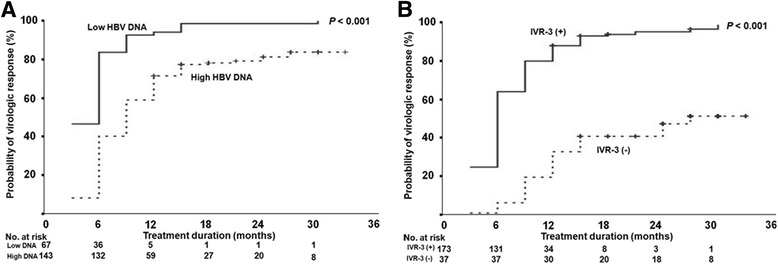
Cumulative virologic response (VR) rate according to HBV DNA levels and IVR-3. **a** Cumulative VR rates in the patients with high baseline serum HBV DNA levels at 6, 12, 24 and 36 months were 40%, 72%, 80% and 83%, respectively. They were 84%, 94%, 97% and 100%, respectively, in the patients with low baseline serum HBV DNA levels. **b** Cumulative VR rates in the patients without IVR-3 at 6, 12, 24 and 36 months were 5%, 32%, 46% and 51%, respectively. And in the patients with IVR-3, they were 64%, 89, 94 and 100%, respectively

Sixty seven patients (31.9%) had low baseline serum HBV DNA levels (<6 log_10_ IU/mL), 173 patients (82.4%) showed IVR-3 and 89 patients (42.4%) were negative for HBeAg. The patients with low serum HBV DNA levels, IVR-3 or HBeAg negativity had a significantly higher probability of achieving a VR. In patients with low baseline serum HBV DNA levels, the cumulative VR rates were 84%, 94%, 97% and 100% at 6, 12, 24 and 36 months, which were in contrast with the rates, 40%, 72%, 80% and 83% in patients with high baseline serum HBV DNA levels (*P* < 0.001, Fig. 4a). The cumulative VR rates were 64%, 89%, 94% and 100% in patients with IVR-3 and 5%, 32%, 46% and 51% in patients without IVR-3 at 6, 12, 24 and 36 months, respectively (*P* <0.001, Fig. 4b). A similar pattern was observed according to HBeAg status (*P* < 0.001).

### Virologic breakthrough during long-term therapy

VBT was experienced in 13 patients, with cumulative rates of 1%, 4% and 11% at 12, 24 and 36 months, respectively. There was no significant difference in the cumulative rates between the ETV and TDF groups (2%, 5% and 12% vs. 1%, 4% and 7% at 12, 24 and 36 months, respectively, *P* = 0.333). VBT developed in 9 cases in the ETV group and in 4 cases in the TDF group. The majority (11/13, 85%) of VBT was related to medication adherence. As expected, mutations associated with drug resistance were detected in 2 patients in the ETV group (L180M+ M204V + S202G).

### Adverse effects

No serious adverse effects such as lactic acidosis or increase in serum creatinine levels were reported in any of the patients in either treatment group. No adverse effects leading to discontinuation of therapy occurred during the whole treatment period. During the follow-up period, 6 patients (5.7%) in TDF group and 7 (6.7%) in ETV group were diagnosed with HCC. The incidence of HCC according to the type of treatment was not different. All of them except two patients were already cirrhotic patients before antiviral treatment, the patients diagnosed with HCC were significantly older than without HCC at the beginning of antiviral treatment (54.2 ± 8.5 vs. 45.7 ± 12.0, *P* = 0.014).

## Discussion

The risk of cirrhosis and/or HCC in CHB increases proportionally as serum HBV DNA levels increase [5, 6]. This finding suggests that sustained suppression of HBV replication with anti-HBV therapy may successfully prevent the progression of chronic liver diseases. Thus, the suppression of HBV replication should be considered the most important therapeutic goal for CHB patients in clinical settings. Currently, ETV and TDF are the most commonly used HBV drugs due to their excellent potency and higher genetic barrier. However, there is limited information on the comparative efficacy of these drugs, and only one drug-to-drug comparison study has been conducted thus far [19]. Although there have been, so far, a few studies evaluating the efficacy of ETV and TDF in treatment-naïve CHB, they included only a limited number of patients or only showed a short-term efficacy [20–25]. Futhermore, most previous studies were retrospective and not randomized, some of them showed different baseline characteristics-such as age, gender or liver disease severity. The present study was also not a randomized controlled trial, but the limitation was complemented by using propensity score. The present study enrolled an adequate number of the treatment-naïve CHB patients and followed these patients for a sufficient time period (up to 39 months) to directly compare the efficacy between ETV and TDF. Of note, in this study, the majority of patients had a VR over 90% after 36 months of therapy. Among HBeAg-positive patients, the cumulative VR rates at month 12 in our study (75% in ETV and 83% in TDF) are comparable with previous global multicenter trials of 67% and 76%, respectively. These results were also comparable in HBeAg-negative patients (97% vs. 90% in ETV; 98% vs. 93% in TDF, respectively) [9, 10, 12].

In this study, ETV and TDF showed similar overall antiviral efficacy both for one year and during the long-term follow-up. These results were similar to previous studies [21–23, 25]. However, our subgroup analysis indicated that TDF was significantly better than ETV for suppressing HBV DNA in HBeAg-positive patients, especially with higher HBV DNA levels. There were a few studies which TDF showed more potent antiviral efficacy than ETV in HBeAg-positive patients [24, 26]. When comparing our study with these previous studies, the present study included only treatment-naïve patients and who had been adherent to medication therapy for at least one year. Patient adherence may contribute to achieve comparable overall VR at month 12 and during long-term period in both groups.

Another interesting finding in the present study is the prognostic role of IVR-3, which is a good predictive factor for both short-term and long-term VR. Higher baseline ALT, lower HBV DNA levels and HBeAg-negative status are already well known to be associated with VR. In addition to these factors, our finding suggested that IVR-3 was also able to predict VR very well. In fact, cumulative VR rates in patients achieving IVR-3 was significantly higher than in patients without IVR-3 (100% vs. 51% at 36 months, RR 3.403, 95% CI 1.977–5.849, *P* < 0.001). Surprisingly, the predictive value of IVR-3 was much higher than those of previously known factors, such as HBeAg status and HBV DNA levels. Pragmatically, IVR-3 combined with HBeAg and HBV DNA levels may help to predict long-term VR and determine whether to maintain primary therapy or switch to other regimens.

Multi-center, long-term studies using ETV and/or TDF have shown excellent safety and tolerance with little antiviral resistance (1.2% in ETV and 0% in TDF up to 5 years). In our study, there was no serious clinical adverse reaction in the two treatment groups. However, even though the patient had achieved VR, HCC was developed in 5.7% of patients receiving TDF and 6.7% of ETV. Considering the risk of developing HCC is appears to be greatest among individuals with the highest serum HBV DNA levels, the introduction of nucleos(t)ide analogues has contributed to reduce the risk of HCC. The reason why the incidence of HCC was higher than previous studies was that the present study included a large number of cirrhotic patients. Cirrhosis has been well known risk factor for HCC. Actually, all patients diagnosed with HCC in this study were cirrhotic patients, except two patients. Our finding confirmed that old and cirrhotic patients should be carefully undergone surveillance for HCC regardless of achieving VR.

It should be considered that poor medication adherence is a major cause of suboptimal response and/or VBT that tends to be increasingly prevalent in proportion to the duration of therapy [27–31]. In the current study, overall cumulative VBT rates were 1%, 4% and 11% at 12, 24 and 36 months, respectively, which was primarily related to medication adherence. Only two patients showed resistance in the ETV group.

Our study has some limitations. First, the study was retrospective and non-randomized. However, we adjusted this shortcoming by using propensity score to minimize the influence of the baseline characteristics. This process made our outcomes to be more reliable. Second, in the process of matching the patients, the sample size was decreased and the follow-up period was shortened. Nevertheless, each group included over 100 patients and the follow-up period was up to 39 months. This is comparable with the most recently published, randomized controlled trial to compare treatment efficacy of ETV and TDF in treatment-naïve CHB patients [32]. Third, the study may not be generalizable to other ethnic groups because we included only Asian patients (Koreans) who were infected by mainly HBV genotype C.

To date, randomized controlled or well matched comparative studies regarding the efficacy of TDF or ETV in treatment-naïve CHB patients were very rare. Therefore, it is necessary to conduct direct comparisons of a prospective nature with larger numbers of patients and longer period of treatment.

## Conclusions

TDF and ETV showed comparable efficacy and safety during 3 years in treatment-naïve CHB patients. We found that IVR-3 was predictive factor on long-term efficacy in addition to HBV DNA levels and HBeAg status. The present study suggested that TDF might be the more potent option in a subgroup of HBeAg-positive CHB, especially with higher HBV DNA levels. VBT occurred with similar rates in both groups, and was primarily the result of medication nonadherence. Old and cirrhotic patients should be carefully undergone surveillance for HCC regardless of achieving VR.

## Abbreviation

ALT: Alanine aminotransferase
anti-HBe: Antibody to hepatitis B e antigen
anti-HBs: Antibody to hepatitis B surface antigen
CHB: Chronic hepatitis B
ETV: Entecavir
HBeAg: Hepatitis B e antigen
HBsAg: Hepatitis B surface antigen
HBV: Hepatitis B virus
HCC: Hepatocellular carcinoma
IVR-3: Initial virologic response at 3 months
TDF: Tenofovir
VBT: Virologic breakthrough
VR: Virologic response
